# Effects of Continuous Theta Burst Stimulation to the Dorsolateral Prefrontal Cortex on Attention to Emotional Stimuli: A Randomized Controlled Trial

**DOI:** 10.3390/brainsci15121328

**Published:** 2025-12-13

**Authors:** Katerina Konikkou, Aimé Isdahl-Troye, Maria Sikki, Kostas Fanti

**Affiliations:** Department of Psychology, University of Cyprus, Nicosia 2109, Cyprus

**Keywords:** transcranial magnetic stimulation, theta-burst stimulation, dorsolateral prefrontal cortex, emotional processing, emotional attention

## Abstract

**Background/Objectives**: The use of theta-burst stimulation (TBS) over the dorsolateral prefrontal cortex (DLPFC) to modulate cognitive function is gaining increasing interest, since it is more time-efficient than standard repetitive transcranial magnetic stimulation. However, the impact of TBS protocols on specific cognitive processes, such as emotional attention, remains understudied. This study aimed to examine the differential effect of continuous TBS (cTBS) over the left and right DLPFC on the facilitation of attention towards emotional (i.e., pleasant and distressing) versus neutral stimuli. **Methods**: In this randomized controlled trial, ninety-one healthy young adults were randomly allocated to receive either real or sham stimulation over the right or left DLPFC (i.e., left/right real cTBS and left/right sham cTBS), and then completed a computerized dot-probe task that included distressing, pleasant, and neutral images. **Results**: Participants who received real cTBS showed slower response times to probes replacing neutral images compared to emotional images, whereas no differences were found between stimuli in the sham conditions. No hemisphere-dependent effects were observed for distressing or pleasant images, suggesting that cTBS modulated attentional performance in a comparable manner when administered over the left or right DLPFC. **Conclusions**: These findings contribute to the literature on emotional attention, underscoring the role of DLPFC in attentional control, which is a valuable cognitive target for advancing the design and implementation of cTBS protocols.

## 1. Introduction

The limited processing capacity of visual perception requires the allocation of attention to stimuli more relevant to one’s current behavior [1]. Two main attentional systems facilitate the processing of visual information. The first one involves exogenous, involuntary processes driven by the perceptual salience of stimuli, and the second accounts for the endogenous, voluntary mechanisms guided by the intentions of the observer [2,3]. These two systems are functionally distinct, but their neural foundations are interconnected [4,5,6]. While the salience of the stimuli competes for neural representation in sensory brain regions [7], the frontoparietal networks filter information and guide attention based on both internal and external goals [1,4,8]. These networks are directly implicated in the voluntary allocation of attention but also play an important role in redirecting the attentional focus towards unexpected stimuli with special salience, which ultimately allows for environmental adaptation.

On this, the intrinsic meaning of emotionally charged stimuli seems to place them in a prominent position within the ‘salience map’. As such, substantial empirical research has shown that emotional stimuli may disrupt the functioning of the attention system, eliciting a rapid orienting response [9], that can interfere with the processing of non-emotional cues [10,11]. Subcortical networks, which include the amygdala, are thought to be implicated in this pre-attentive evaluation process of salient emotional stimuli [12,13] that enhances sensory processing and facilitates the reallocation of attention toward emotionally significant information [11,12]. In parallel, existing evidence indicates that the interaction between the amygdala and frontal areas of the brain, including the dorsolateral prefrontal cortex (DLPFC), may modulate emotional processing [11,14,15]. There is evidence suggesting that (1) the DLPFC is activated when there is an inhibition of the response to emotional stimuli in the amygdala [16,17], and that (2) an existing (direct or indirect) communication between amygdala and DLPFC (along other prefrontal regions) explains emotion regulatory processes [14,18]. Accordingly, other studies have shown that reduced activity in the DLPFC might be related to increased emotion-driven behavior [19,20]. The complementary nature of these findings provides unison support to consider the neuromodulation of this region as an important target to alter emotion processing. Nevertheless, the advancement of research in this direction is coupled with an ongoing debate on functional asymmetry of brain hemispheres in the processing of emotions [21], which raises questions about the specific role of the prefrontal cortex.

The idea of analogous regions in the two hemispheres working on distinct aspects of cognitive function came from classical studies of brain damage. This line of work suggested that the right hemisphere has a dominant role in the processing of all emotions [22,23]. However, this view was not always supported in the literature (e.g., [24]), since additional research found alternative explanations, such as the asymmetrical specialization of the frontal cortex depending on the emotional characteristics of the stimuli [25,26]. Based on this evidence, two main hypotheses have been proposed. The first one is the valence hypothesis [27,28,29], which suggests that the left frontal cortex processes positive and approach-related emotions, whereas the right frontal cortex processes negative and avoidance-related emotions. The other hypothesis is the asymmetric inhibition model, which is built on the valence hypothesis but proposes that a right-lateralized executive control inhibits positive or approach-related distractors, while left-lateralized control inhibits negative or withdrawal-related distractors [25]. From an integrative perspective, evidence suggests that both hemispheres might contribute to emotional processing with complementary roles [30,31,32], but still much remains to be understood about their specific contribution.

Of note, existing evidence around the DLPFC hemispheric specialization on emotional attention came from studies that used a variety of techniques to assess brain functioning. In recent years, studies using non-invasive brain stimulation have been introduced in the field, as these techniques can selectively modulate cortical activity and thus effectively explore changes in attentional processes [33,34,35,36]. For example, repetitive transcranial magnetic stimulation (rTMS) has shown promise in selectively modulating neural activity with minimal side effects [37]. This technology is commonly used in clinical studies on emotional disorders, such as depression [38,39,40] and anxiety [41,42], but also it showed promising effects in the management of substance and behavioral addiction [43], as well as to symptoms related to borderline personality disorder [44,45] or eating disorders [46,47]. Although the beneficial effects of rTMS on emotional and behavioral symptoms are gaining increased attention, the specific mechanisms through which it generates these effects are not yet clear. Because of this, a growing number of studies are now aiming to explore the cognitive mechanisms that may underlie the behavioral enhancement observed with rTMS (e.g., [48,49]).

Several studies explored the effect of brain stimulation over the DLPFC on attention and emotional processing in clinical and non-clinical populations, finding that rTMS over the DLPFC might modulate attentional engagement toward emotional content [17,50,51]. Regarding the right hemisphere, high-frequency rTMS over the DLPFC might strengthen top-down control over aversive stimuli [52], but facilitate attention toward threatening information in participants with higher baseline anxiety [53]. Moreoever, Zwanzger and colleagues showed that low-frequency rTMS over the right DLPFC resulted in a preference for fearful expressions in healthy participants [54]. Regarding the left hemisphere, Sagliano and colleagues found that low-frequency rTMS over the DLPFC was linked to a disengagement bias towards threatening stimuli in high anxious participants, but to an avoidance response in those who showed low anxiety [51]. Additionally, Balconi & Caravesio found that low-frequency rTMS over the left DLPFC resulted in a pattern of reduced responsivity to facial expressions of happiness [55]. It is worth mentioning that the behavioral or cognitive changes resulting from rTMS might be attributable to the modulation of diverse brain networks interconnected with the local site of stimulation [56]. Accordingly, a review by Guse and colleagues indicated that rTMS over the DLPFC may indirectly affect connected areas related to attention and emotion [57]; however, the specific mechanisms underlying cognitive improvement with rTMS remain unclear.

More recently, theta burst stimulation (TBS) protocols, which were designed with the intention of enabling a more rapid, time-efficient, induction of functional and activity changes in cortical regions [58], are emerging as a promising alternative to traditional rTMS. Whereas a typical rTMS session can last up to 37 min, TBS applications take between 40 and 190 s to reach comparable effects [59,60]. For instance, clinical sham-controlled trials are providing evidence for similar antidepressant effects of TBS and traditional rTMS over the DLPFC [60,61]. Promising results such as these are inspiring new research focused on exploring the potential of TBS as an intervention method but also as assessment tool [19,62,63]. TBS protocols include two main modalities: intermittent TBS (iTBS) and continuous TBS (cTBS). Current literature supports that both protocols over prefrontal regions can modulate performance on measures of executive functions in non-clinical populations [63]. The cTBS protocol is usually associated with a deterioration effect on task performance while iTBS is associated with an enhancing effect. Specifically, the systematic review and meta-analysis conducted by Lowe and colleagues [63] on healthy adults suggested that iTBS might have enhancing task-dependent effect (e.g., on working memory), whereas cTBS seemed to reliably attenuate performance in different measures of executive functions, especially when targeting the left prefrontal cortex. Upon these findings, Ngetich and colleagues [64] added that cTBS either on the right or left prefrontal cortex have the potential to influence cognitive functioning, with (at a first sight) “paradoxical” improvement on planning and decision-making (in line with the idea of “addition-by subtraction” by Luber & Lisanby [65]). This suggests that the disruption of the competing processes activated for performing a task might lead to a reorganization of cognitive resources that facilitates performance. In line with this “selective” disruption of cognitive function, Wyczesany and colleagues [66] suggested that cTBS over the right DLPFC could compromise cognitive control during reappraisal without altering emotional perception. In turn, Schmaußer et al. [67] showed that cTBS over left dlPFC decreased resting vagally mediated heart rate variability (vmHRV), which is a marker of autonomic linked with cognitive-affective regulation. Overall, this evidence posits cTBS as a value tool for designing experimental models in healthy adults that might help disentangling the role of DLPFC on top-down control and basic emotional processing.

Initial evidence for the impact of TBS on emotional attention facilitation of visual content comes from work applying cTBS over the right DLPFC [68]. In this study, cTBS effects were examined during an emotional Go/NoGo task involving happy and fearful faces while recording electroencephalogram (EEG) oscillations. Participants receiving real versus sham cTBS over the right DLPFC exhibited decreased alpha power oscillations in response to happy stimuli, which is an indicator of increased cortical activity [69]. Another study suggested that cTBS over the right DLPFC resulted in enhanced occipital–parietal brain activity in response to negative images [70]. These findings seem to contradict the valence hypothesis and the asymmetric inhibition model and instead suggest that frontal asymmetry alone may not account for the observed effects of prefrontal cortex involvement in emotional processing. However, more studies targeting both the left and right DLPFC are needed to examine these hypotheses.

In relation to the neurophysiological mechanisms underlying the effects of TBS protocols, various explanations are currently under examination, including the interference with cortical rhythms [62,71,72] and the influence on synaptic connections though a possible effect on neurotransmission system [58,73]. However, the overall behavioral and physiological evidence of TBS effects in non-motor areas is still inconsistent, and research is needed to verify and fully understand these effects [56,74].

The purpose of the present study was to examine the role of the left and right DLPFC in modulating attentional responses to emotional stimuli, specifically pleasant and distressing images. Previous research has addressed this question using neuroimaging techniques, but such approaches do not allow researchers to draw conclusions about the involvement of specific brain regions in cognitive processes [67]. To address this limitation, it is worth considering the potential of cTBS, as one single session using this protocol provides the opportunity to induce a rapid, but temporary and reversible, alteration of a targeted brain area [75]. As such, cTBS offers a valuable tool for investigating the functional contributions of cortical regions to cognitive function, including potential hemispheric asymmetries in emotional processing, but also for exploring compensatory mechanisms that help overcoming those temporary alterations to brain activity [75]. Under the assumption that cTBS may inhibit (more than facilitate) brain activity [58], for instance in the DLPFC, there is an opportunity to assess how attentional responses are affected when the neural activity of one hemisphere is suppressed, and as a result the contralateral hemisphere assumes a more dominant role. As said, despite the potential of this approach, the number of studies examining emotional attentional asymmetries using cTBS remains limited, with fewer studies comparing the effects of inhibitory stimulation over both the left and right DLPFC [76,77].

A widely used paradigm in studies of visual attention is the dot-probe task, which aims to capture attention facilitation and bias towards emotional stimuli. This task relies on the principle that individuals tend to respond more quickly to a probe when it replaces a stimulus that has previously captured their attention. Consistent with this suggestion, some studies have shown faster responses to probes replacing distressing images compared to neutral ones (e.g., [78]), and in some cases, similar facilitation effects have been observed for pleasant stimuli (e.g., [79]). The dot-probe task can show emotionally evocative images from the International Affective Picture System (IAPS; [80]), which were used in diverse samples [81,82,83]. IAPS images can typically evoke higher levels of arousal than emotional faces, making them particularly suitable for investigating emotional attention in both general and subclinical populations.

Based on evidence showing that emotional stimuli facilitate attention, and that cTBS can modulate emotional processing [68,76,77], we hypothesized that participants receiving inhibitory cTBS over the DLPFC would exhibit faster reaction times to probes replacing pleasant and distressing images, relative to a sham condition. Furthermore, drawing from the valence hypothesis and the asymmetric inhibition model, we expected hemisphere-specific effects: inhibition of the left DLPFC might enhance attentional facilitation toward distressing stimuli (due to the dominance on the right hemisphere), whereas inhibition of the right DLPFC might facilitate attention toward pleasant stimuli (for opposite reasons). Because of the mixed results from the cTBS literature in relation to brain lateralization of emotional processing, and due to the novelty of this protocol for examining emotional attention in healthy participants, our study was mainly exploratory. However, we did expect that findings can contribute to a better understanding of how emotional attention is shaped by prefrontal mechanisms, and thus to provide new insights for research aimed at developing (or improving existing) clinical interventions to modify attentional biases associated with affective-related difficulties [84,85].

## 2. Materials and Methods

### 2.1. Participants

Based on a power analysis with moderate effects (f = 0.25) with α = 0.05 and power = 0.95, ninety-one participants (32 Males: Mage = 21.60, SD = 1.60; 59 Females: Mage = 20.82, SD = 1.45) were recruited from a community sample in Cyprus. These participants were all healthy, medication-free, right-handed and had normal vision (for computer tasks). None of the participants had any established risk factor to rTMS following the screening Transcranial Magnetic Stimulation Safety Questionnaire (e.g., cardiac pacemakers, epilepsy, and use of drugs) [86]. Further exclusion criteria included the history of psychiatric/neurological disorders, such as epilepsy, head trauma and migraine.

### 2.2. Research Design

The study followed a double-blind, randomized, sham-controlled design. Participants were randomly assigned to one of four stimulation conditions: (1) real cTBS on the left DLPFC, (2) real cTBS on the right DLPFC, (3) sham cTBS on the left DLPFC, and (4) sham cTBS on the right DLPFC. Response times (RTs) to distressing, pleasant, and neutral stimuli, as well as the prioritization of attention toward emotional versus neutral stimuli (outcome variables), were assessed using the dot-probe paradigm following a single session of brain stimulation. The experimental group (i.e., between-subjects factor) and the valence of the stimuli (i.e., within-subject factor) were treated as independent variables.

### 2.3. Study Procedure

The study was approved by the Cyprus National Bioethics committee. Participants were recruited via advertisement and word-of-mouth. All of them provided a written informed consent for their participation in this experiment and were informed about the TMS procedures. As reported in Table 1, there were twenty-four participants assigned in the real left group (Female, F = 17 and Male, M = 7) and twenty-two in the sham control group (F = 13 and M = 9). The real right group included twenty-three participants (F = 16 and M = 7), and twenty-two participants were assigned to the right sham group (F = 13, M = 9). Of note, a single-session of rTMS is usually not found to acutely affect mood in healthy volunteers [87], but it has been reported that state anxiety prior to stimulation (perhaps related to expectations concerning the rTMS procedures) affects cognitive-affective responses [53,88]. Thus, all participants were asked to complete two well-validated questionnaires assessing anxiety (State-Trait Anxiety Inventory—Trait Subscale, STAI-T [89]) and depression (Beck Depression Inventory, BDI-II [90]) to establish that experimental groups did not differ on these measures. As reported in Table 1, the four experimental groups were comparable regarding age, depression and anxiety scores.

### 2.4. Experimental Procedure

The experimental procedure was driven by the experimenter, and both male and female participants were pseudo-randomly assigned to a color-group according to the order of arrival at the lab: ‘blue’, ‘green’, ‘yellow’ and ‘orange’. Thus, the experimental conditions were unknown to the participant. Participants were told that they belong to the color-group indicated by the card they had chosen and that they will be presented with an attention computerized task after receiving a short cTBS session (40 sec). Immediately after stimulation, participants were comfortably seated in front of a 19’ computer screen (1024 × 768) at an approximate distance of 80 cm. Participants first completed a training phase (12 pairs) of the task, to allow themselves to familiarize with the overall procedure. The dot-probe task stimuli were presented in random order, and the E-Prime 2.0 software (Psychology Software Tools, Pittsburg, PA, USA) was used for stimuli presentation and data collection.

cTBS Protocol. Coil positioning was determined using standardized coordinates from the EEG International 10–20 system [91], with F4 corresponding to the right DLPFC stimulation target and F3 corresponding to the left DLPFC. The location and orientation of each participant’s coil placement was indicated on a nylon cap that participants wore throughout the single stimulation session. A figure-of-eight focal coil (70 mm diameter) was used. The coil was held in a fixed position by a mechanical arm and oriented so that the induced electric current flowed in a posterior–anterior direction. Stimulus intensities were set at 70% of active motor threshold (AMT). Previous experiments that used cTBS found significant inhibitory effects using as stimulation intensity 70% of the AMT [92,93]. In addition, we chose to use 70% intensity to prevent intense facial muscle twitching during stimulation [94]. Each TBS session burst consisted of 3 pulses at 50 Hz, with each train being repeated every 200 ms (5 Hz) for 40 s (600 pulses). It has been shown that this stimulation paradigm suppresses cortical excitability. The number of pulses per participant used in the present study is consistent with the prototype protocol [95].

Sham Protocol. Sham stimulation was delivered using an identical with the real Magstim figure-of-eight focal sham coil (70 mm diameter) that produced the same stimulation noise. Sham Magstim figure-of-eight coil have been found to induce nearly zero electric-field under the coil’s center [96,97], which make it a credible placebo procedure. The coil was held in a fixed position by a mechanical arm and oriented so that the induced electric current flowed in a posterior–anterior direction. Stimulus intensities were the same as the real stimulation condition and the same positioning procedure was followed using the 10–20 system for all participants.

Emotion Dot-Probe Task. The dot-probe task is a common laboratory paradigm used to index attentional bias for emotional stimuli at early stages of information processing [98]. It provides a quick, convenient, and inexpensive index of emotional responsiveness. The emotional pictures version of the task used in the current study presents a series of picture pairs of distressing (e.g., crying child), neutral (e.g., book) and pleasant (e.g., smiling baby) emotional content using images primarily taken from the IAPS [80]. Images were selected based on previous studies to tap distress and pleasant content [82,83]. This task consists of 1 block of practice stimuli (12 picture pairs) followed by 3 experimental blocks, each containing 12 picture pairs. Each picture presentation had three sequential components: (1) a 500 millisecond image of fixation cross appearing in the center of the screen, (2) a 500 millisecond simultaneous presentation of one of three potential picture pairings: neutral-neutral, pleasant-neutral and distress-neutral, with stimuli centered and located above or below the location of the fixation cross, and (3) a second image of fixation cross appearing in either the top or bottom picture location. Participants were instructed to respond as fast as they could and after every trial they selected a key on the keyboard that corresponded to the location on the screen (up or down) where the dot-probe appeared. If no key was pressed within 5000 milliseconds, the response was recorded as incorrect.

The number and location of picture stimuli were counterbalanced across test trials in order to ensure that an equal number of emotional and neutral stimuli appeared in both top and bottom locations. Additionally, there were equal number of emotional and neutral stimuli that were replaced versus not replaced by a dot-probe stimulus. The primary dependent measure for the current study was an attentional facilitation index. This facilitation index was calculated by subtracting the average RT (latency) to either dot-probes replacing emotional pictures in distress-neutral or pleasant-neutral pairs from the average latency to probes replacing neutral stimuli in the various neutral-neutral picture pairs (12 pairs each). Thus, this procedure resulted in two facilitation indices relating to distressing and to pleasant images. To control for potential location effects, such as an attentional preference for the top or bottom location of the screen, the following formula was used to calculate the facilitation indices by comparing neutral with emotional probes in the same location: Facilitation = 1/2 × [(Neutral Only/Dot-probe Up − Emotional Up/Dot-probe Up) + (Neutral Only/Dot-probe Down—Emotional Down/Dot-probe Down)]. In this formula, “emotional” refers to either distressing or pleasant images, depending of the facilitation index being calculated. Consistent with prior uses of the task [99], incorrect responses and RTs less than 100 milliseconds were not included in the calculation of facilitation indices. Facilitation scores greater than three standard deviations above or below the mean were truncated to 3 SDs. Given that emotional pictures typically facilitate allocation of attention, participants were generally expected to respond more quickly to probes replacing emotional images, resulting in a facilitation index to pleasant or distressing images.

### 2.5. Plan of Analysis

A series of repeated measures ANOVA models were performed using IBM SPSS 20.0. As a preliminary step, the first of these models explored the differences in RTs depending on the valence of the stimuli (neutral, distressing or pleasant). This model was performed prior to the main analyses, in order to test the potential of the emotional cues to effectively retain the attention in the dot-probe task [33,100]. The next model examined the effects of the cTBS conditions (to either the right or the left DLPFC) on attention allocation (i.e., facilitation index), in comparison to the sham stimulation conditions. The within-subject variable was the valence of the images, with the stimulation conditions as the between-subjects variable. A facilitation index for each of the two emotional stimuli (distressing and pleasant) was calculated by subtracting the average RT to dot-probes replacing emotional pictures (distress and pleasant) from the average RT to probes replacing neutral stimuli. Partial eta squares (η2 = 0.01–0.06 small effect size, η2 = 0.06–0.14 medium effect size, η2 > 0.14 large effect size, [101]) are reported in the text. Interaction effects are depicted in figures along with 95% confidence intervals. Pairwise comparisons among conditions (post hoc analyses) were adjusted to Bonferroni correction in order to minimize Type I error.

## 3. Results

The means and SDs corresponding to the RTs of each study condition are presented in Table 2.

### 3.1. Effects of Emotional Stimuli on RTs

A significant within-subject main effect was found for the valence of the stimuli, *F*(2, 88) = 77.46, *p* < 0.001, η^2^ = 0.47. Results indicated that participants responded faster to distressing (*M* = 428.39, *SE* = 8.32; *p* < 0.001) and pleasant images (*M* = 422.64, *SE* = 7.93; *p* < 0.001) compared to the neutral ones (*M* = 472.57, *SE* = 8.20).

### 3.2. Effects of Stimulation Conditions on RTs

**Figure 1:**
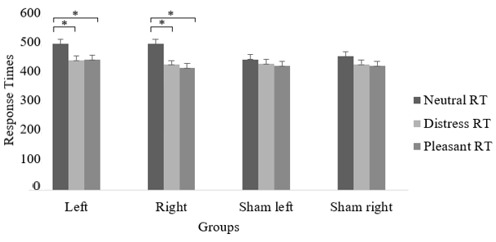
Average Response Times (RT; in ms) to neutral, distressing and pleasant images across DLPFC hemispheres and cTBS condition. * indicates significant difference (*p* < 0.05) between emotional stimuli in each stimulation condition (i.e., Left = real cTBS on left DLPFC, Right = real cTBS on right DLPFC, Sham left = sham cTBS on left DLPFC, Sham right = sham cTBS on right DLPFC).

The main effect for the stimulation condition was not significant, *F*(3, 87) = 0.69, *p* = 0.55, η^2^ = 0.02. However, there was a significant Emotion × Stimulation interaction *F*(6, 174) = 7.208; *p* < 0.001, η^2^ = 0.20. Specifically, results showed that participants in the real cTBS conditions responded faster to emotional (both pleasant and distressing) compared to neutral stimuli. The same effect was identified for both the left and right stimulation, but not for the sham conditions (see Figure 1).

### 3.3. Attentional Facilitation Indices

**Figure 2:**
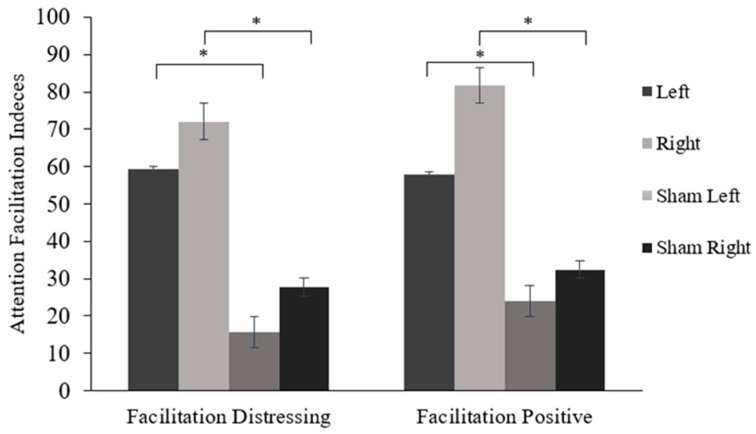
Response time differences among emotional and neutral stimuli (facilitation index) for distressing and pleasant images, considering DLPFC hemisphere and cTBS condition. * indicates significant difference (*p* < 0.05) in facilitation index of distressing and pleasant images between stimulation condition (i.e., Left = real cTBS on left DLPFC, Right = real cTBS on right DLPFC, Sham left = sham cTBS on left DLPFC, Sham right = sham cTBS on right DLPFC).

Results showed a significant within-subject effect for attentional facilitation indices, *F*(1, 90) = 5.58, *p* < 0.05, η^2^ = 0.06). Pleasant stimuli elicited a higher facilitation effect (*M* = 49.94, *SE* = 5.01; *p* < 0.05) compared to distressing stimuli (*M* = 44.18, *SE* = 4.80). A between-subjects effect for type of stimulation was also identified, *F*(3, 87) = 8.38, *p* = 0.001, η^2^ = 0.22. On average, participants who received real left and right cTBS showed higher overall attention facilitation to positive and distressing stimuli compared to both sham conditions. No significant interaction was observed between the facilitation effect and the stimulation conditions, *F*(3, 87) = 1.27, *p* = 0.29, η^2^ = 0.04. However, post hoc comparisons using Cohen’s d effect sizes revealed that real left cTBS (*M* = 50.41, *SE* = 7.91; *p* < 0.05; *d* = 0.49) and real right cTBS (*M* = 76.25, *SE* = 7.87; *p* < 0.05; *d* = 0.61) resulted in significantly larger facilitation effects to pleasant images compared to both sham left (*M* = 23.91, *SE* = 8.09) and sham right conditions (*M* = 32.45, *SE* = 7.74), respectively, with moderate effect sizes (see Figure 2). Additionally, both real cTBS left (*M* = 52.71, *SE* = 7.84; *p* < 0.05; *d* = 0.42) and real cTBS right (*M* = 67.12, *SE* = 7.52; *p* < 0.05; *d* = 0.46) conditions resulted in a significantly larger facilitation effect to distressing stimuli compared to both sham left (*M* = 15.64, *SE* = 8.02) and sham right (*M* = 27.66, *SE* = 7.68) conditions (see Figure 2).

## 4. Discussion

The present study aimed to explore the role of the DLPFC in emotional attention by applying a single-session cTBS over either the right or left hemisphere. To this end, participants received either real or sham cTBS prior to completing an emotional dot-probe task that included pleasant, distressing, and neutral images. The main measure was attentional facilitation to emotional stimuli, indexed by the differences in RTs to emotional compared to neutral stimuli [99]. Results revealed that participants who received real stimulation, regardless of hemisphere, tended to respond slower to neutral images than to emotional ones. This response pattern was not observed in the sham conditions. Accordingly, higher facilitation effects were observed for pleasant and distressing stimuli after applying real cTBS when comparing to sham conditions. In view of this, the observed changes in the allocation of attention might be attributable to the impact cTBS might have on the cortical activity in the DLPFC. This outcome, while preliminary, adds to the still modest cumulus of literature exploring the causal role of DLPFC activity in visual attention and perception of emotions (e.g., [17,52]), and particularly in research applying a task-paradigm that considers diverse emotional contents (e.g., [33,76]).

That presupposed change we observed in the attentional pattern after cTBS also encourages the use of this protocol to deepen the understanding of several clinical conditions characterized by an imbalance in the oriented response towards emotional vs. neutral content [102]. Maladaptive attentional biases toward emotional cues have been linked to various psychological disorders, such as anxiety [103,104,105], depression [106], and borderline disorders [107], to name a few. The cTBS protocol used in this study might have simulated in human participants without clinical levels of anxiety and depression what occurs (to some extent) in DLPFC functioning when the responses to neutral information slow down respect to normality, and the emotional stimuli receive attentional priority. About this, it must be stressed that the facilitation of attention to emotional stimuli in our study did not seem to derive from a faster orienting response to emotional cues per se, as no differences in RTs for emotional images were observed between the real and sham stimulation groups. In fact, the main difference involved the facilitation index, which pointed to higher attention orienting to distressing and pleasant stimuli among participants receiving real stimulation, compared to those in the sham condition. This finding was evident irrespective of stimulation in the left or right DLPFC. Although these findings are important, it is not clear why the application of cTBS over the DLPFC slowed down participants’ orienting responses to non-emotional stimuli, which resulted in the identified between group differences in the facilitation index. Nevertheless, such findings might point to the critical role of the DLPFC in regulating both emotional [54,108] and cognitive functioning [65,109].

One possibility is that neural activity in the DLPFC may relate to interference inhibition [48,49], which is essential to maintain efficiency in task-performance. Thus, increased activation of the DLPFC could enhance the deployment of cognitive resources (e.g., processing speed) needed to accomplish a demand in a given context [110]. Conversely, some studies suggest that cTBS over the DLPFC can simulate a situation of low cognitive control that leads to more emotion-driven behaviors [19]. Building upon results of this exploratory study, we hypothesize that a possible reduction in the activity of the DLPFC might interfere with decision-making processes, delaying responses to less engaging content (i.e., neutral stimuli), while leaving responses to emotional stimuli relatively unaffected due to their automatic salience. Evidence from Keuper and colleagues [70] and Cao and colleagues [68] supports this notion that cTBS to the DLPFC can enhance stimulus-driven attention toward emotional cues. These enhancing effects could be mediated through exogenous (bottom-up) mechanisms, which may remain operational even when top-down control is temporarily suppressed, for example, by the action of cTBS in the DLPFC. Thus, current findings might suggest that a presupposed DLPFC inhibition does not necessarily impair or bias attention towards emotional content, but rather shifts the balance toward more automatic, stimulus-driven processing. The preserved response to emotional stimuli observed in our study might reflect this dynamic adaptation of the human brain.

To broaden its scope, this work aimed specifically to examine the lateralization of emotional attention in the DLPFC, considering stimuli that elicit either pleasant or distressing emotions. No preliminary evidence for lateralized effects of DLPFC stimulation were found in this exploratory study. Thus, our results differ from previous work that supports either the right hemispheric hypothesis [23], or the hemispheric asymmetry hypothesis [27,77] and the asymmetric inhibition model [25] in the processing of emotional content. Instead, current findings indicated that both right and left DLPFC regions might contribute to attention allocation, regardless of the emotional valence of stimuli. This approach supports previous work claiming that both hemispheres may contribute, in a complementary manner, to emotional processing [30,31,32], which also provides evidence for the brain’s capacity for functional compensation. Therefore, one possibility is that neural plasticity mechanisms may have allowed contralateral regions to maintain attentional performance following localized inhibition, influencing facilitation to emotional stimuli differentiated on valence.

Our findings must be interpreted in light of several limitations. First, there is increasing evidence of the need to consider inter- and intra-individual variability in the frontal brain function when assessing TMS effects [74,111,112]. In this sense, the participant cognitive and affective state during the brain stimulation session may modulate the resulting effects on the stimulated area [113,114,115]. Although we accounted for the potential interfering effects of emotional symptoms, following previous findings regarding the sensitivity of TMS protocols to baseline levels of depression and anxiety (e.g., [53,88]), findings would have been enhanced if neuroimaging techniques were employed to test and control for interindividual variability in brain functioning [41,111]. Second, this study has applied cTBS over the DLPFC to assess the potential effect of a (supposed) temporary alteration in this region on a cognitive function of interest (i.e., emotional attention). Following this rationale, we consider that the application of a single session of cTBS in a community-based sample is justified. Nevertheless, future work should test the durability of cTBS effects by including follow-up assessments. In addition, the identified cTBS effects cannot be generalized to actual clinical populations, and future studies that include participants with attentional bias are necessary to explore the clinical relevance of our findings. Third, the random assignment of participants to experimental (real cTBS) and control (sham cTBS) conditions allowed between-group comparisons in the performance of the emotional dot-probe task after brain stimulation. The inclusion of an analogous pre-test task was dismissed in the study design to avoid familiarity or learning effects. However, this has limited the exploration of within-person, intra-individual changes, which would have contributed to a more comprehensive interpretation of the results. Fourth, this study was based on the assumption that the effects of cTBS on the cortical activity in the DLPFC are inhibitory. However, it is important to acknowledge that such inhibitory effects of the cTBS have not always been sufficiently reported in regions outside the motor cortex. Consequently, researchers such as Hussein & Friedmann are emphasizing the need for caution when making inferences on the relationship between brain with cognition and behavior [56]. Five and finally, the dot-probe task remains a widely used paradigm to measure emotional attention, but its reliability has been recently questioned [116,117]. Future research should consider including eye-tracking data in addition to RT to enhance the reliability of attentional markers.

Besides these limitations, this study is one of the few that have compared the effect of cTBS to the right and left DLPFC, after accounting for their analogous sham control conditions. We believe that through this design, the study findings can contribute to the literature exploring brain asymmetry in emotional processing. Although the findings did not reveal a direct effect of the DLPFC on the processing of emotional stimuli, we hypothesized this might respond to the potential mechanisms that preserve attention to these stimuli despite potential local alterations in this region, both in the right and left hemispheres. Such findings do not only support previous literature on the intrinsic value of emotional stimuli but also point to compensatory mechanisms that help preserve key cognitive functions, such as emotional attention. However, we do acknowledge that brain networks that sustain emotional attention may involve the coordination of the DLPFC with subcortical structures such as the amygdala, which are responsible for the prioritization of the focus of attention towards salient emotional stimuli [118,119].

In addition, slowing the top-down processing in response to irrelevant information, while maintaining attention to emotional stimuli, might have therapeutic value. The clinical utility of cTBS has been previously explored from the standpoint of its possible inhibitory effects on cognitive function, with evidence suggesting that the combination of cTBS and iTBS can enhance the treatment of depression [120]. In addition, cTBS on the right DLPFC could contribute to the treatment of naturalistic phobias (i.e., spiders) through a disruption effect on the reconsolidation of fear memory [121]. Based on our findings and previous work using cTBS in healthy adults [66], we suggest that cTBS might be an interesting tool to complement therapies aimed at facilitating the access to emotional experience or awareness while reducing excessive cognitive control and emotional avoidance; two maladaptive forms of emotional regulation linked to anxiety and depression [122,123]. Moreover, the combination of TBS and cognitive therapies, such as attentional bias modification interventions [124,125,126], could boost both top-down and bottom-up driven changes in attention. This may be of interest as an approach to treating conditions involving a heightened affinity for, or difficulty disengaging from, negative stimuli that generate emotional distress. Further research is needed to optimize such therapeutic protocols and validate findings in clinical contexts.

## 5. Conclusions

This study provides initial evidence that cTBS over DLPFC modulates attentional responses differently to neutral and emotional stimuli. Slower responses to neutral images might suggest enhanced attentional engagement towards emotionally salient cues, potentially driven by reduced top-down control. As such, these results support the idea that non-invasive neuromodulation may offer a promising avenue for assessing and treating emotional processing deficits. Future studies should explore the effect of TBS in the enhancement (or reduction) of attention as a therapeutic component in behavioral and cognitive interventions aimed at altering attentional bias toward emotional stimuli.

## Figures and Tables

**Table 1:** Demographic information per stimulation condition.

	N	cTBS Left	cTBS Right	Sham Left	Sham Right		
**Total**	91						
Male	32	7	7	9	9		
Female	59	17	16	13	13		
							
		M (SD)	M (SD)	M (SD)	M (SD)	*F*(1,90)	*p*
Age		20.00 (1.16)	21.17 (1.74)	21.13 (1.78)	22.00 (3.23)	1.04	0.37
BDI-II		3.29 (2.40)	3.09 (2.12)	4.34 (2.06)	4.03 (3.02)	0.24	0.88
STAI-T		39.71 (10.36)	37.37 (8.99)	37.33 (9.02)	38.05 (7.66)	0.35	0.79

**Table 2:** Average Response Times (RT; in ms) to neutral, distressing and pleasant images across cTBS condition (real/sham) and DLPFC hemispheres (left/right).

	Emotion	M (RT)	SD (RT)
Real Left	Distressing	440.70	87.27
	Pleasant	442.09	94.34
	Neutral	499.95	98.34
Real Right	Distressing	426.15	58.97
	Pleasant	415.08	62.13
	Neutral	503.51	76.31
Sham Left	Distressing	424.63	64.77
	Pleasant	416.35	58.91
	Neutral	440.27	59.66
Sham Right	Distressing	425.69	92.25
	Pleasant	422.90	96.32
	Neutral	453.61	82.90

## Data Availability

The dataset presented in this article is not readily available due to privacy and ethical restrictions.

## Abbreviations

The following abbreviations are used in this manuscript:

DLPFC	Dorsolateral Prefrontal Cortex
rTMS	Repetitive Transcranial Magnetic Stimulation
TBS	Theta-Burst Stimulation
cTBS	Continuous Theta-Burst Stimulation
iTBS	Intermittent Theta-Burst Stimulation
EEG	Electroencephalogram
AMT	Active Motor Threshold
RT	Response Time
M	Mean
SE	Standard Error
